# Our first year and beyond

**DOI:** 10.1038/s42003-019-0295-8

**Published:** 2019-01-31

## Abstract

*Communications Biology* celebrates its 1 year anniversary of publishing advances across the biological sciences. Here we review our first year of publishing and look forward to what we hope to achieve in the years to come.

We launched *Communications Biology* in January 2018 to provide a forum for all biologists to publish high-quality research, reviews and commentary in any area of the biological sciences^1^. While many specialist journals exist across the various disciplines within biology, as do broad-scope journals publishing work of general interest across the natural sciences (such as *Nature* and *Nature Communications*), we felt that there were still few journals open to publishing a wide range of scientific articles without a requirement for broad, general interest beyond the immediate specialist field.

Based on the number of submissions received to date, it seems that the biology research community agreed. When we ask authors their motivation for choosing to submit their paper to our infant journal, a frequent answer is that they did not feel their work was suitable for any particular specialist journal because the subject matter is multidisciplinary. Other common reasons for submission to *Communications Biology* include its open access publishing model, our focus on early-career researchers, and affiliation with the Nature Research group of journals.

Whatever the reason, we are immensely grateful to researchers who have chosen to publish their discoveries in our pages. As of our publishing anniversary of January 22nd, 2019, we have published more than 240 primary research articles and 8 review and commentary articles. Many of our articles have been covered in the popular media and we are confident that all will inspire further advances in their respective fields. As part of our anniversary celebrations, our in-house editors have chosen a few articles that they personally found inspiring, which we have highlighted in a collection available at our homepage. In addition to our editor’s picks, we will be publishing Comment articles in which previous authors provide an update on their research since publishing their paper in *Communications Biology*. Look for these over the next few weeks.

We launched *Communications Biology* with a number of goals in mind. First and foremost is of course our commitment to publishing high-quality, innovative research across the life sciences. In addition, we sought to make *Communications Biology* a journal by and for the community of biologists. Our editorial board of active researchers is part of this goal. We have also sought to highlight the contributions of individual researchers through initiatives such as our early-career researcher spotlights^2^, travel grants to early-career researchers (which we sponsor together with our sister journals, *Communications Chemistry* and *Communications Physics*), and our reviewer of the month program. As part of an effort to get more researchers involved in the review process, particularly those who feel they have not had ample opportunity, we have also placed a permanent call for reviewers on our journal website (https://www.nature.com/commsbio/referees).

Another goal of the journal is to promote transparency in publishing. We are therefore excited to begin publishing reviewer reports and author replies alongside our research articles this year. Authors of papers submitted from January 1, 2019 will have the option to participate in this transparent peer review program. We will also begin publishing the Nature Research Reporting Summary document, as is already done at other Nature Research journals^3^, for all papers accepted to the journal starting this year.

Our first year of publishing has been very exciting, but we are more excited still for what the future will bring. We will continue to build upon our success by experimenting with ways to improve our author and reader experience and engage with our reviewers. But we have ambitions beyond simply being a great place to publish and read the latest research. Among our many goals is to further our mission of promoting diversity and inclusion in everything we do. This means ensuring diversity among our editorial staff and editorial board—in terms of gender, ethnicity, geographical location, and subject area expertise—and among our commissioned authors, reviewers, and research topics. We also hope to provide a venue to publish research and commentary that advances the goal of bringing the benefits of scientific research to a diverse community of people, particularly in the areas of human genomics, agriculture, and epidemiology.

An example of work that fits this aim is the recently published Review by Melinda Mills and Charles Rahal^4^. Their scientometric review of genome-wide association studies makes clear the overrepresentation of people of European ancestry in genetic studies, among both participants and the researchers themselves. Within their recommendations to the scientific community, they highlight the need to prioritize inclusion at multiple levels—including ancestral, geographical, and environmental—to increase the diversity of genetic data available. They also call on researchers to involve and mentor scientists from underrepresented ancestry and geographical regions, as well as early-career researchers and women, to help build the skills and capacity to conduct large-scale genetic studies independently.

We at *Communications Biology* want to take this opportunity to invite submissions of articles that advance our diversity goals. We invite research in all areas of biology that foster collaboration with and inclusion of scientists from diverse backgrounds—including geographic location, ethnicity, gender, and career stage—and that addresses biological questions affecting the non-Western world. An example of the type and quality of work we are inviting is shown in a paper published today in which researchers from UC Davis and the International Institute of Tropical Agriculture in Kenya were able to improve the stress resistance of plantains using CRISPR/Cas9^5^. All submissions will be assessed for novelty, scientific advance, and potential impact on their respective fields.

We invite research in all areas of biology that foster collaboration with and inclusion of scientists from diverse backgrounds and that addresses biological questions affecting the non-Western world.

We look forward to publishing these and many more exciting advances in the years to come.
